# Patterning of graphene using wet etching with hypochlorite and UV light

**DOI:** 10.1038/s41598-022-08674-3

**Published:** 2022-03-16

**Authors:** Minfang Zhang, Mei Yang, Yuki Okigawa, Takatoshi Yamada, Hideaki Nakajima, Yoko Iizumi, Toshiya Okazaki

**Affiliations:** 1 CNT Application Research Center, National Institute of Advanced Industrial Science and Technology (AIST), Higashi 1-1-1, Tsukuba, Japan; 2 Nanomaterials Research Institute, National Institute of Advanced Industrial Science and Technology (AIST), Higashi 1-1-1, Tsukuba, Japan

**Keywords:** Chemistry, Materials science

## Abstract

Graphene patterning via etching is important for enhancing or controling the properties of devices and supporting their applications in micro- and nano-electronic fields. Herein, we present a simple, low-cost, and scalable wet etching method for graphene patterning. The technique uses hypochlorite solution combined with ultraviolet light irradiation to rapidly remove unwanted graphene areas from the substrate. Raman spectroscopy, atomic force microscopy, scanning electron microscopy, and optical microscopy results showed that well-patterned graphene with micrometer scale regions was successfully prepared. Furthermore, graphene field effect transistor arrays were fabricated, and the obtained devices exhibited good current–voltage characteristics, with maximum mobility of ~ 1600 cm^2^/Vs, confirming the feasibility of the developed technique.

## Introduction

Graphene, a one-atom-thick sheet of carbon atoms arranged in a honeycomb-like structure, is the thinnest, strongest, and most conductive material of both electricity and heat^1–3^. Graphene has attracted widespread attention for its potential use in the next generation of electronic devices and other fields ranging from biosensors to energy storage systems^4–8^. For these applications to be realized, well-defined architectures at micrometer and nanometer scales will need to be determined.

Various patterning approaches have been proposed, including bottom-up fabrication methods and top-down etching processes involving lithography^9^. Bottom-up fabrication includes the spatially selective synthesis of graphene arrays by patterning of catalysts^10,11^, or selective decomposition of silicon carbide (SiC) to form graphene arrays^12^. Selective synthesis methods suffer from a variety of limitations, including the use of harsh synthesis conditions, complicated steps, demanding large-scale preparation, and structural limitations^9^. By contrast, top-down patterning by etching of designed areas is expected to be an effective strategy. The techniques of photolithography and electron beam lithography combined with dry etching by oxygen or plasma^13–16^, reactive ion etching^17^, helium ion ablation^18^, and laser ablation^19,20^ have been used for patterning graphene on substrates. Results indicate high pattern resolution down to 10 nm, and high alignment accuracy^18^. However, these dry etching processes suffer from a variety of limitations including high cost due to expensive equipment, and complex or inconvenient processing steps that are time-consuming and difficult to scale up. Additionally, the high power of plasma or ion beams may induce defects in patterned graphene as well as the substrate, which may damage the electronic properties.

By contrast, wet etching processes are generally simpler, use low-cost equipment, and are easier to scale up for industry production^21^. Wet etching also has the advantage of being less damaging to the etched object, which expands the range of its practical applications. However, wet etching methodology has not yet been developed for graphene patterning. It is believed that the stable graphitic structure of graphene makes it difficult to remove or dissolve by chemical agents in solutions.

Recently, we found that carbon nanotubes (CNTs)^22–25^ and graphene oxide^26^ could be degraded by hypochlorite, and the degradation rates could be enhanced significantly by combining with ultraviolet (UV) light irradiation. In the present work, we found that sodium hypochlorite (NaClO) could completely degrade monolayer graphene films, which can be used as a wet etchant for graphene patterning. Compared with the dry etching, the wet process is simpler, more affordable, scalable, and easier to control due to the ambient reaction conditions. The patterning process consists of three steps: protection with a gold (Au) cover through convenient electronic beam (EB) evaporation, wet treatment with hypochlorite, and stripping of the Au cover. Using this process, different graphene pattern designs were successfully prepared without damaging the structures, as evidenced by Raman spectra mapping, optical microscopy, scanning electron microscopy (SEM), and atomic force microscopy (AFM). Moreover, as a demonstration, graphene field effect transistor (FET) arrays were fabricated, and the obtained devices exhibited good current–voltage characteristics, confirming the feasibility of the developed technique. The results expand our knowledge about the degradation of graphene in solution, and provide a simple and practical method for electronic processing of graphene, which could expand the application potential of this material for use in micro- and nano-electronic devices.

## Results

### Degradation of monolayer graphene

**Figure 1:**
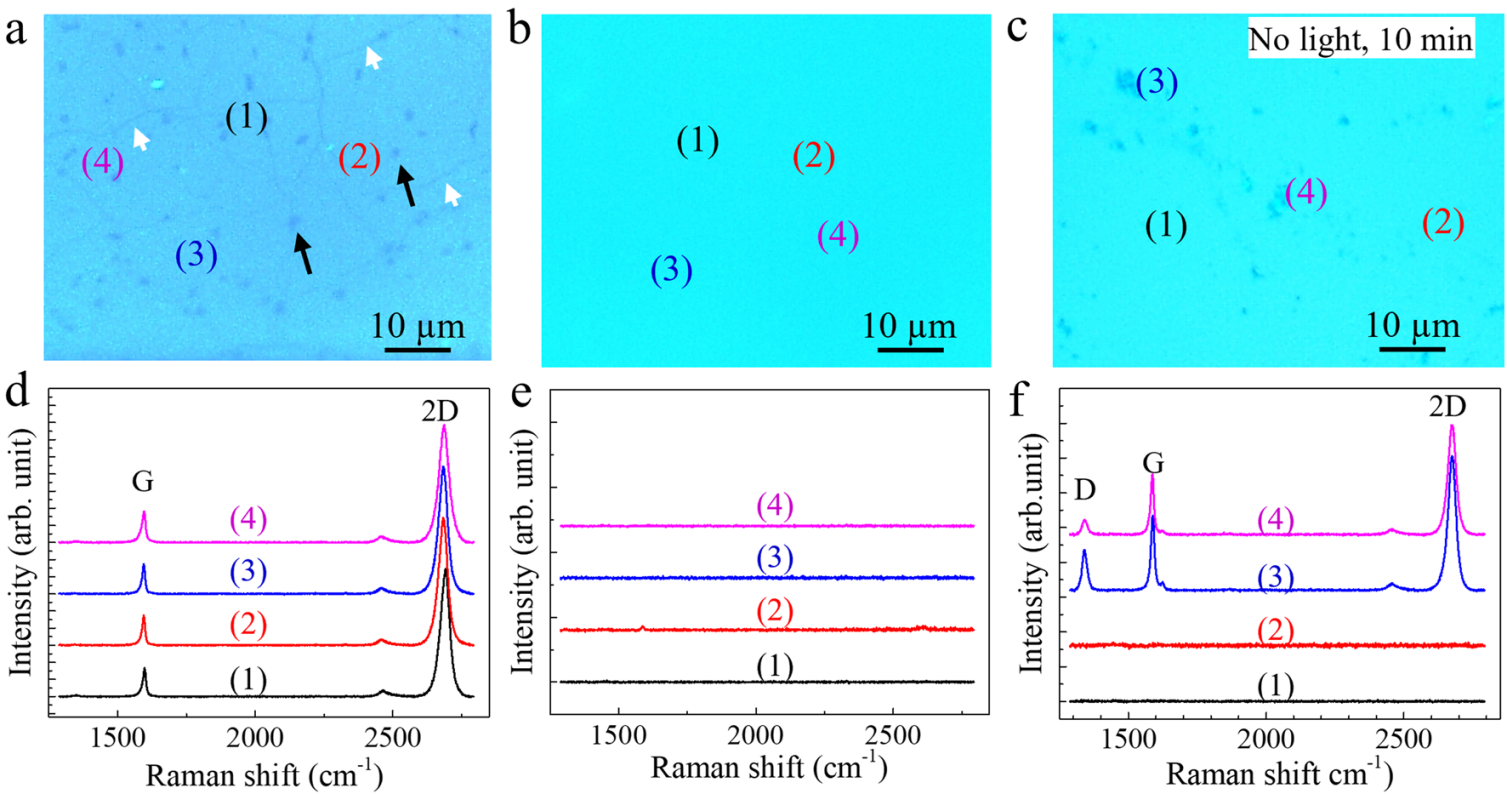
Degradation of graphene by bleach solution (NaClO, 6 wt%). (**a**) Optical microscopy images of monolayer graphene film on SiO_2_/Si substrate. Lines (indicated by white arrows) and dark spot-like areas (indicated by black arrows) represent the domain boundaries and regions with few graphene layers, respectively. Images acquired after treatment with bleach solution assisted by UV light irradiation were captured after 5 min (**b**), and without light irradiation after 10 min (**c**). Raman spectra (**d**, **e**, and **f**) for locations (1, 2, 3, and 4) correspond to images (**a**, **b**, and **c**), respectively.

The monolayer graphene film, used as purchased, which was produced by chemical vapor deposition and transferred onto SiO_2_/Si substrate (labeled graphene/substrate hereafter) via a sacrificial copper film. The graphene film had a smooth surface with some lines and dark spot-like areas (Fig. 1a) that were assumed to be the connected boundaries of graphene domains^27^ and regions with few layers^28^, respectively. Raman spectra from four random points on the film-covered substrate were almost the same, indicating uniformity of the graphene film, and all spectra had G-band and 2D-band peaks at ~ 2683 and ~ 1590 cm^-1^, respectively. The full width at half maximum (FWHM) was ~ 37 cm^-1^, and the peak intensity ratio (2D/G) was > 5, which indicated monolayer graphene (Fig. 1d)^28^. When the graphene/substrate was immersed in bleach solution with a NaClO concentration of ~ 6 wt% and then irradiated by UV light for 5 min, the color of the surface of the graphene/substrate appeared brighter and lighter than before treatment. Optical microscopy images revealed no footprints of graphene such as boundaries or spot-like areas (Fig. 1b). The disappearance of graphene was also confirmed by Raman spectra measurements; the absence of peaks indicated that there was no graphene remaining at any points on the treated film (Fig. 1e). On the other hand, when the graphene/substrate was treated with the same bleach solution but without light irradiation for 10 min at room temperature, the graphene film was not completely cleaned; some graphene patches (3 and 4 in Fig. 1c) remained on the surface of the substrate. Raman spectra measurements confirmed that the features were indeed graphene, and the blank areas were cleared spaces (Fig. 1f). This result indicates that UV light irradiation enhanced the graphene degradation rate.

To further prove the complete degradation of graphene by NaClO solution, we treated graphene nanoplatelets, consisting of short stacks of graphene sheets with a few layers, with NaClO (6 wt%) solution (SI, Figure SI-1). Following treatment, the black graphene dispersion became a colorless transparent solution, and the amount of graphene in solution was decreased to almost zero after treatment in an incubator (70 °C) for 6 h, indicating that graphene degraded completely, consistent with our previous results for CNTs^23–25^.

### Patterning graphene by wet etching with hypochlorite

**Figure 2:**
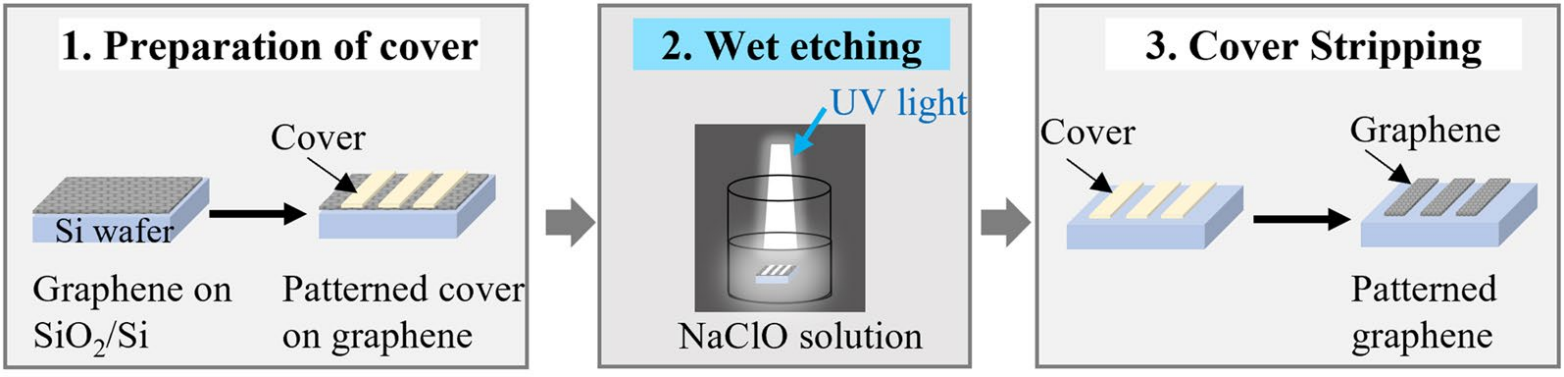
Schematic illustration of wet etching by hypochlorite for graphene patterning.

The process for patterning graphene using NaClO as an etchant generally consists of three steps: preparation of the protecting cover, wet etching, and stripping of the cover (Fig. 2). The protective cover with a designed pattern was prepared using metals or photoresistor polymers, as employed for dry processes, for which lithography is a convenient technique. The protective cover used in this study was obtained by EB evaporation of Au with various designed patterns on the surface of the graphene/substrate. After the protective Au cover was prepared, samples of the graphene/substrate were treated with a bleach solution (NaClO = 6 wt%) and irradiated by a UV light for 5 min (Fig. 2). After treatment, the sample was removed immediately, rinsed with water, and immersed in gold etching solution containing KI/I_2_ to strip the Au cover^29^.

**Figure 3:**
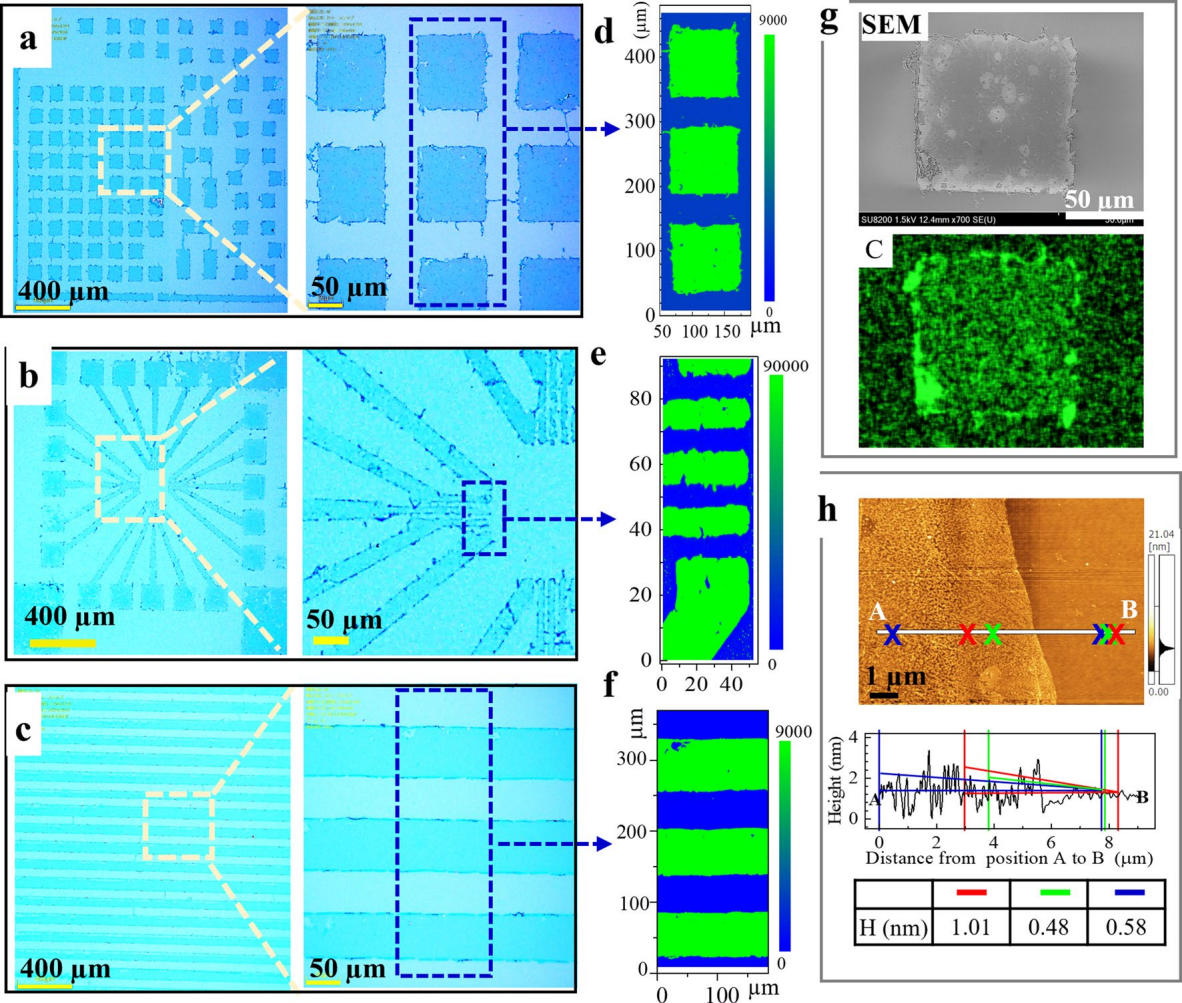
Three typical patterned graphene arrays. Optical microscopy images show close-up views of different areas of the different patterned graphene arrays on Si-wafers obtained by NaClO etching after gold cover removal (**a**, **d**, and **c**). Raman mapping of areas (marked as blue squares in **a**, **b**, and **c**) is shown for 2D peak intensities at ~ 2683 cm^-1^ (**d**, **e**, and **f**). (**g**) SEM image from one of the patterned square areas in (**a**) alongside the elemental carbon (C) mapping image. (**h**) Atomic force microscopy (AFM) topographic image for one of the patterned graphene ribbon edges in image (**c**), and the corresponding height profiles for the line from position A to position B (bottom).

The results showed that well-patterned graphene arrays were successfully prepared using the above process. Three typical graphene, namely squares (Fig. 3a), radiation shapes (Fig. 3b), and ribbons (Fig. 3c), were observed by optical microscopy, with widths of 10–100 µm and no obvious defects. Raman mapping of the peak intensities of 2D-bands at ~ 2683 cm^-1^ indicated that the patterning of graphene arrays in all three patterns was successful (Fig. 3d, e, and f). SEM images and corresponding energy-dispersive X-ray spectra (EDS) mapping of elemental carbon also confirmed that the patterned area was graphene (Fig. 3g), and the thickness of graphene in prepared patterns was about 0.5–1.0 nm as measured by AFM (Fig. 3h).

To further clarify the structural damage induced by wet treatment, a large area (565 × 660 µm) was evaluated for patterned graphene arrays by Raman mapping of the D-band peak at 1350 cm^−1^, which corresponds to the amount of disorder or defects in graphene, while the G-band at 1590 cm^−1^ is indicative of the graphitic structure, as is the typical graphene peak of the 2D-band. The results showed that the intensities of D-bands, as well as G-bands and 2D-bands, were almost uniform for all measured arrays (SI, Figure SI-2). Mapping of the intensity ratio of D and G peaks (D/G) also showed no increase in the domains and edges of graphene arrays. In addition, the intensities of G-band and 2D-band peaks at a few edges were slightly stronger than at other areas, indicating overlap of graphene in these locations, which was confirmed by the results from SEM. SEM images of one patterned graphene square showed that a small part of the patterned graphene edge was rolled up (SI, Figure SI-3). Moreover, images for elemental mapping of carbon (C) and oxygen (O) obtained by EDS measurements showed that O did not increase obviously following treatment at both edges and domains of graphene, but C at the rolled edge was increased slightly compared with the rest of the domain area (SI, Figure SI-3).

### Fabrication of the graphene FET

**Figure 4:**
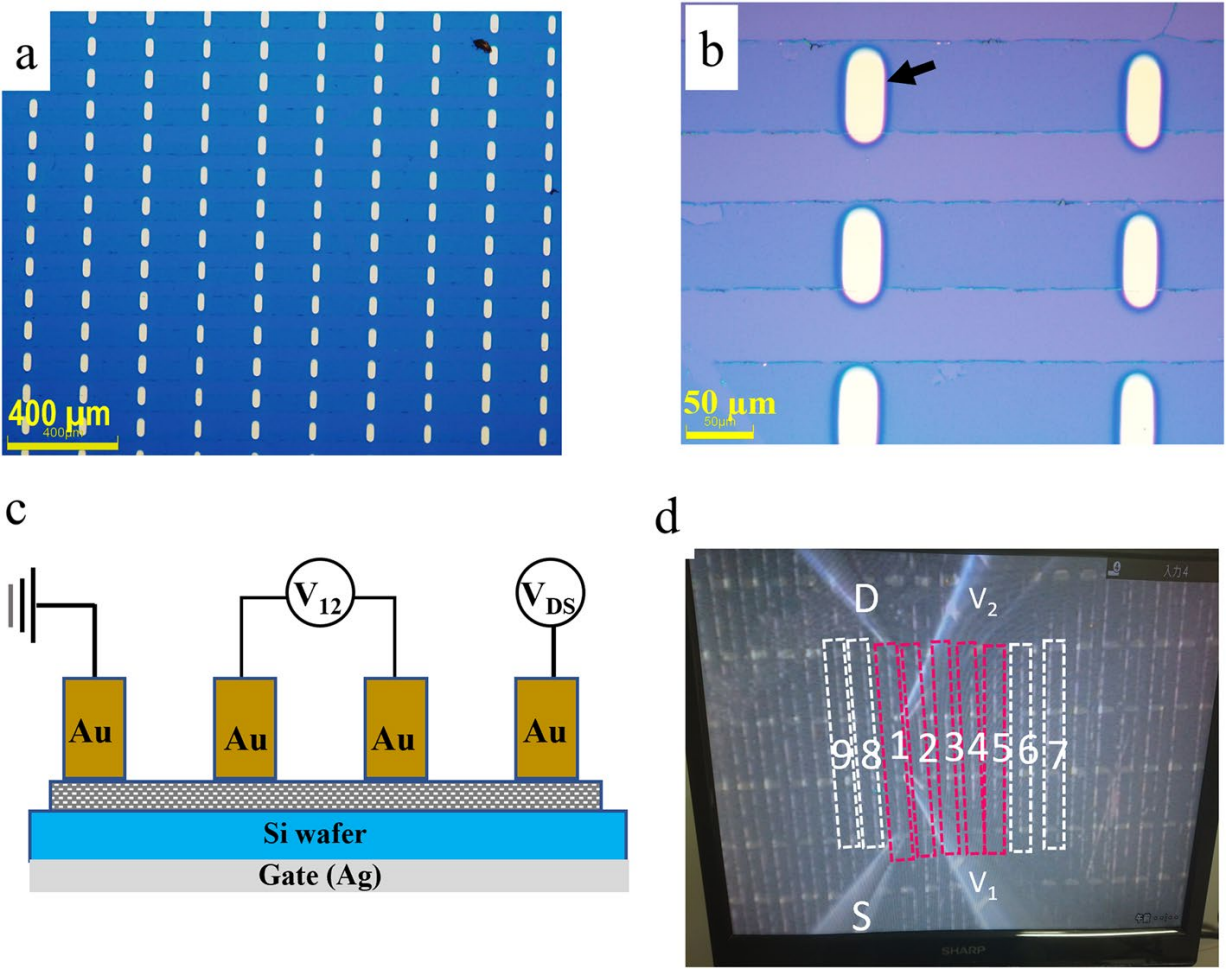
Illustration of the fabricated graphene field effect transistor (FET). The optical microscopy image (**a**) and close-up view (**b**) are shown for patterned graphene arrays with an Au electrode (light yellow rectangle) on the Si-wafer. The schematic diagram shows the back-gate FET (**c**) and a photograph of the obtained device in the measurement chamber (**d**).

To explore device applications, FET arrays were fabricated as a demonstration. To contact the electronic probes on the patterned graphene arrays obtained using the above process, Au and Cr were deposited on the graphene arrays as electrodes at widths of 60 ± 10 µm; the space between the two electrodes was about 200 ± 10 µm (Fig. 4a and b); and the back gate was prepared by coating Ag paste on the back of the substrate (Fig. 4c). The resulting channel length and width of the patterned graphene FET were 200 ± 10 and 60 ± 10 µm, respectively.

**Figure 5:**
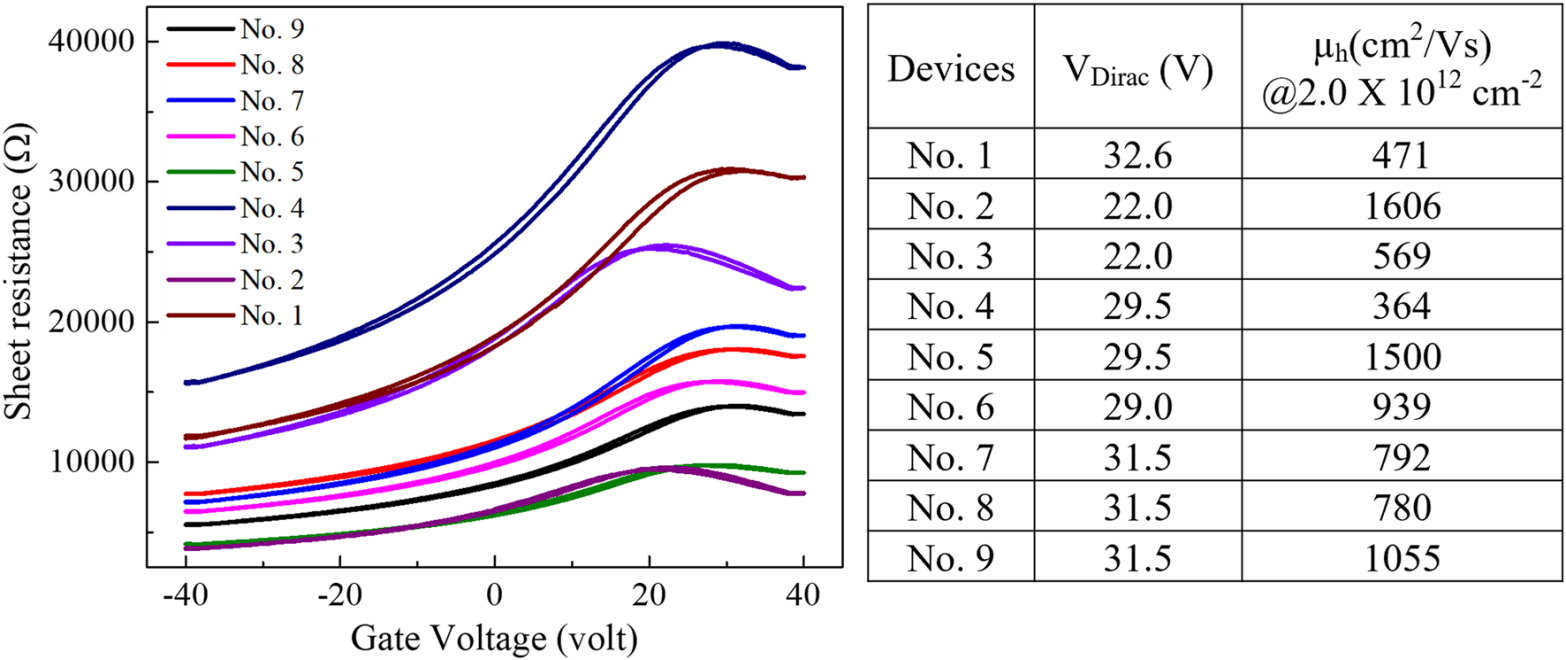
Characteristics of the fabricated graphene FET. The transfer characteristics curves of electronic resistance spanning a gate voltage from -40 to 40 V are shown for nine devices (left), along with the Dirac points and electron mobilities (right). The ratio of length to width for graphene channels was presumed to be 3, and n = 2.0 × 10^12^ cm^-2^.

The drain current vs. gate-source voltage (*I*_*D*_*-V*_*GS*_) characteristics of the obtained FET was investigated by measurements made using 4-point probes in a vacuum chamber (2 × 10^–2^ Pa) at room temperature (Fig. 4d). The results of nine arbitrary devices showed that the electric resistance changed as back-gate bias V_g_ varied from − 40 to 40 V (Fig. 5, left). The minimum conductivity points in transfer characteristics, also known as Dirac points, were clearly observed from the curves (Fig. 5, right). The positive Dirac points indicated that the devices were p-type in nature.

## Discussion

It is known that graphene and graphite can be oxidized by oxidizing acids such as HNO_3_^30^, H_2_SO_4_/HNO_3_^31^, and KMnO_4_/H_2_SO_4_^32^ to form graphene oxide or graphene dots, but studies on the complete degradation or elimination of graphene have not been reported, except for combustion. In the present study, we found that a monolayer graphene film could be degraded completely and rapidly using NaClO solution, a household bleach, when assisted by UV light irradiation. The rapid elimination of graphene encouraged us to use the approach for patterning of graphene.

Crucial to the rapid etching of graphene in this study was UV light irradiation. Although the mechanism of graphene degradation by NaClO assisted by UV irradiation is not fully understood, it is known that UV light accelerates the deposition of NaClO to generate photooxidants, such as HO^**∙**^, O^**∙**−^, and Cl, which possess very strong oxidation ability^33^. These oxidants react rapidly with graphene, as occurs in water treatment^34^ and the purification of fuel gas^35^. The complete oxidation of graphene by NaClO is believed to follow the general reaction formula C_graphene_ + 2NaClO → 2NaCl + CO_2_, similar to CNTs ^23^. In the case of light irradiation, the super-reactive oxygen species would preferentially react with defective carbon, and oxidation would then spread out, leading to the removal of domains and eventually of the whole graphene film (SI, Figure SI-4). This is supported by the graphene film degradation results showing that some domains of graphene were removed preferentially before complete elimination (SI, Figure SI-5).

Another important factor when using bleach is the inclusion of a surfactant such as an alkylether amine oxide (AO) in addition to NaClO. To explore the effects of the surfactant, we treated graphene films with NaClO solution (~ 6 wt%) with or without addition of AO (1% N,N-D-dimethyldodecyamine-N-oxide) instead of bleach solution. The results showed that etching of graphene for patterning was possible through treatment with light/NaClO solution with or without surfactant, but addition of surfactant made the removal faster and more thorough (Figure SI-6). We believe that the surfactant increased the permeability of NaClO and the affinity of hydrophobic graphene in aqueous solution. However, additional studies using different concentrations of AO surfactant as well as other types of surfactants are clearly needed.

In addition, we found that NaClO at a concentration of 6 wt% (SI, Figure SI-6) could remove graphene more rapidly than at a concentration of 2.26 wt% (SI, Figure SI-5) and 1.1 wt% (SI, Figure SI-1), indicating that a higher concentration was more effective for degradation of graphene, consistent with the results for CNTs^25^.

The process for patterning of graphene employed in this study was simple and convenient, and involved the preparation of a protective cover using an established method, and very simple wet etching using a cost-effective etchant (bleach) and inexpensive equipment. Moreover, the wet process was easily controllable and scalable because the reaction was performed by simply immersing the graphene/substrate in bleach solution at room temperature followed by UV light irradiation. For successful patterning, it is important to prepare a suitable protective cover, which can be prepared from a noble metal such as Au (Fig. 3) or by using a negative photoresistor (SI, Figure SI-7). The high precision of the prepared protective cover would yield patterned graphene with similarly high precision. If the protective cover is prepared by Au EB evaporation via a readymade metal mask, the cover will be in the micrometer scale due to current limitations of the technique. In addition, the mild conditions for wet etching and the gentle removal of the protective cover could decrease the roughness of the edges of the pattered graphene.

In addition, the characteristics of the patterned graphene as well as the electrical properties of the FET are also affected by the quality of the original graphene film, especially regarding defects, layers, and the adherence state of graphene to substrates such as SiO_2_/Si. The FET devices fabricated herein from the commercially available graphene samples exhibited ambipolar current–voltage characteristics. Although it is necessary to take electrode configuration into account when estimating hole mobility (µ_h_), the highest value obtained herein was ~ 1600 cm^2^/Vs, confirming the feasibility of our wet etching technique. Notably, the gate voltage at Dirac points (V_Dirac_) and the mobilities for different devices varied from 29 to 31 V and from 400 to 1600 cm^2^/Vs, respectively. This might be due to the different properties of local original graphene structures and/or interactions with substrates. Although the value of the charge mobility in the graphene FET obtained in this study was not outstanding compared with previous reports^36^, the results indicate that our wet etching process is applicable to the patterning of graphene for fabrication of electronic devices.

The p-type properties of these FET devices were consistent with previous reports for monolayer chemical vapor deposition (CVD) graphene on SiO_2_^37–40^. It is probable that the degree of graphene coupling to the SiO_2_ substrate might induce hole doping^37^. We believe that p-type conduction was not due to the etching process using aqueous NaClO solution. The obtained hole mobilities of monolayer CVD graphene were in the range of reported data, which were calculated from channels of almost the same size^38–40^. To ensure good reproducibility, a high-quality and homogeneous graphene structure as well as appropriate adherence to the substrate are extremely important, alongside the patterning process.

In conclusion, we found that monolayer graphene could be rapidly and completely eliminated by etching with a household bleach assisted by UV light irradiation. The etching process was simple, cost-efficient, and scalable, which enabled the successful patterning of graphene with various fine-tuned patterns. The FET arrays fabricated as a demonstration exhibited good performance in terms of ambipolar current–voltage characteristics and high hole mobility, indicating the feasibility of the developed wet etching technique. Further improvement in the graphene patterning process may be possible by preparing a high-precision protective cover at sub-micrometer scale, and by developing more appropriate conditions for etching graphene on different substrates. We believe that the wet etching technique proposed herein could be useful for various processes using graphene in electronic applications.

## Methods

### Degradation of graphene by sodium hypochlorite

Monolayer graphene films with a thickness of ~ 0.345 nm were used as purchased (Catalog no. 773700, Sigma-Aldrich, Tokyo, Japan). Films were deposited on SiO_2_/Si by direct CVD via a sacrificial copper film. Graphene on the SiO_2_/Si substrate was 1 × 1 cm in size, with a coverage > 95%. The thickness of substrate was ~ 525 µm with a coating of thermal oxide (SiO_2_) of ~ 300 nm on both wafer sides. NaClO solution used in this study was a household bleach solution (Kitchen Power Bleach, Lion Hygiene, Tokyo, Japan) with a NaClO concentration of ~ 6 wt% and AO surfactant (1–5 wt%). This bleach solution was used directly without dilution within 3 months after purchase. Besides the standard bleach solution, NaClO solutions with 1.1 wt%, 2.26 wt%, or 6 wt% were also tested, and were prepared by dissolving NaClO·5H_2_O powder (Wako, Tokyo, Japan) in deionized water.

To investigate the degradation of graphene, the graphene/substrate was immersed in bleach or NaClO solution (~ 5 mL) at room temperature, and then irradiated by a UV light spot (1 cm diameter) using a Lightningcure LC5 light source unit (Hamamatsu Photonics, Hamamatsu, Japan) composed of a Hg-Xe lamp (200 W) and fiber cables. The UV light intensity at 365 nm was ~ 3.5 W/cm^2^, and the wavelength had a range of 240–550 nm.

### Graphene patterning

#### Preparation of the protective cover on graphene film

The protective cover was made by decomposition of Au (thickness = 100 nm) on graphene film through a patterned mask using an EW-100S vacuum EB evaporation instrument (Eiko Corporation, Tokyo, Japan). Two types of patterned mask were tested: one was a photoresistor polymer assisted by lithography (SI, Figure SI-8), which was used for preparation of pattern A (Fig. 3a) and pattern B (Fig. 3b); the other was a custom-made metal (Ni) mask used for designed pattern C (Fig. 3c), which was prepared by the manufacturer (Fuji Semitu Industries, Osaka, Japan) using a traditional metal etching method.

#### Wet etching

After covering with the patterned protective Au film, the graphene/substrate was immersed in bleach solution or NaClO solution and then irradiated by UV light for ~ 5 min. The graphene/substrate was removed immediately, rinsed three times with deionized water, and dried naturally at room temperature blown gently with N_2_ gas.

#### Stripping the protective cover

After wet etching, the graphene/substrate samples were immersed in KI/I_2_ solution^29^ for 10–60 s to remove the Au cover. KI/I_2_ solution was prepared by dissolving 1.6 g KI powder (Wako) and 0.4 g I_2_ (Wako) in 50 mL water. When the yellow pieces of Au cover were floating on the surface, the graphene/substrate was taken out, rinsed three times with deionized water, and dried naturally at room temperature or blown gently using N_2_ gas.

### Evaluation of graphene after wet etching

After treatment or patterning, graphene was first evaluated by optical microscopy using a DSX 510 instrument (Olympus, Tokyo, Japan) with 10× and 50× lenses. Images were captured using bright field (BF) mode, and samples were checked by micro Raman spectroscopy (inVia, Renishaw, UK). Raman measurements were taken at an excitation wavelength of 532 nm. Raman mapping of patterned graphene was performed by measuring the peak intensities for 2D-bands at ~ 2683 cm^-1^, G-bands at ~ 1590, and D-bands at 1350 cm^-1^. SEM using an SU8220 instrument (Hitachi High Tech Corporation, Tokyo, Japan) combined with EDS using a 5060FlatQUAD instrument (Bruker Corporation, Yokohama, Japan) were employed to assess the structure of patterned graphene, and to investigate the elemental distributions by mapping of carbon and oxygen in the graphene arrays. SEM and EDS measurements were performed at an acceleration voltage of 5 kV and an emission current of 10 µA. AFM images were obtained by a Nanosearch scanning probe microscope combined with an optical/laser microscope (Shimadzu, Tokyo, Japan).

### Fabrication and evaluation of the graphene FET array

Patterned graphene ribbon arrays were selected for preparation of FETs. The drain and source electronic probes were prepared using Cr/Au EB evaporation by an EW-100S vacuum EB evaporation instrument (Eiko Corporation) and a custom-made metal (Ni) mask. Cr and Au were successively deposed at a thickness of ~ 20 and ~ 100 nm, respectively.

To assess the performance of fabricated graphene FETs, the patterned graphene ribbons with Au/Cr electrodes were thermally treated at 623 K under an H_2_ atmosphere (40 Pa) for 2 h to remove residues on the graphene surfaces that may otherwise cause resistance. Samples were then transferred to another vacuum system (Thermal Block Company, Kawaguchi, Saitama, Japan) for electrical measurements. The devices were heated to 473 K in the chamber before measuring electrical properties to remove any water molecules adhered to the graphene. *I*_*D*_ as a function of *V*_*GS*_ was measured at room temperature below 2.0 × 10^–2^ Pa using a standard four-probe technique with a 4200A-SCS semiconductor parameter analyzer (Tektronix Inc., Tokyo, Japan). Under a constant drain-source voltage (*V*_*DS*_) of 0.1 V, *V*_GS_ was swept from -40 to 40 V. The hole mobility was calculated using the following equation:

$${\mu }_{h}=\frac{1}{ep{R}_{s}}, n={C}_{ox}({V}_{GS}-{V}_{Dirac})$$

where *R*s is the sheet resistance of the patterned graphene, e is the elementary charge (1.6 × 10^19^ C), C_ox_ is the gate insulator capacitance (t_SiO2_ = 300 nm), and *V*_Dirac_ is the gate voltage at the Dirac point. The mobility at a hole concentration (p) of 2.0 × 10^12^ cm^-2^ is discussed in this study.

## Supplementary Information

Supplementary Information.

## Data Availability

All experimental data that support the findings of this study are available from the corresponding author upon reasonable request.
