# Supplementary material for: Patterning of graphene using wet etching with hypochlorite and UV light
Source: Sci Rep. 2022 Mar 16;12:4541. doi: 10.1038/s41598-022-08674-3 (PMC8927452; doi:10.1038/s41598-022-08674-3)
Supplement: Supplementary file 1 — Supplementary Information. [file 41598_2022_8674_MOESM1_ESM.pdf]

Supplementary information for

Patterning of graphene using wet etching with hypochlorite and ultraviolet light

Minfang Zhang[1]*, Mei Yang[1], Yuki Okigawa[2], Takatoshi Yamada[2]*, Hideaki Nakajima[1], Yoko Iizumi[1], Toshiya Okazaki[1]*

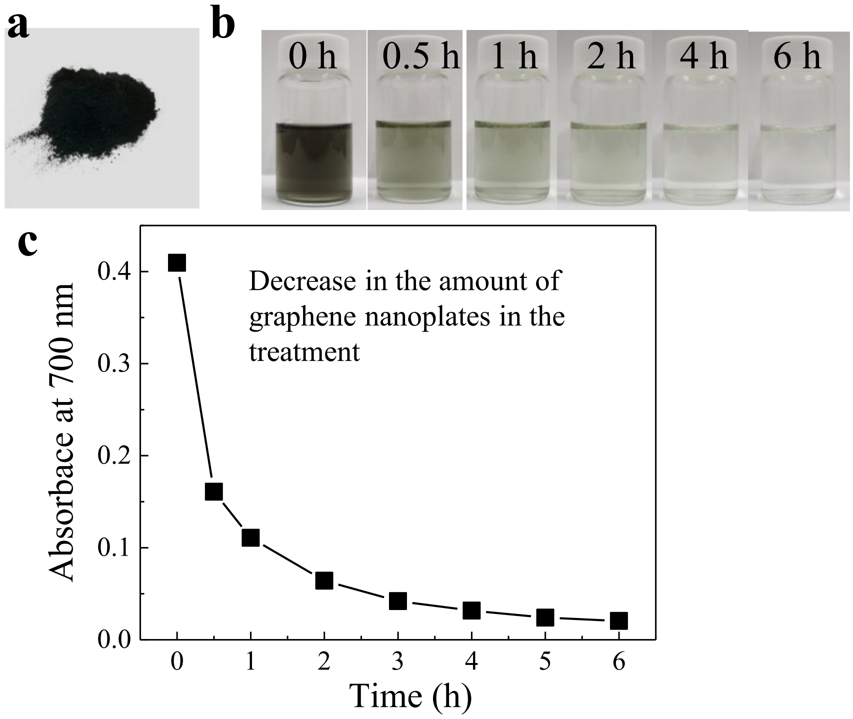

Figure SI-1. **Demonstration of the complete degradation of graphene by NaClO solution.** (a) Photos of graphene nanoplatelet (GN) powder (900407, Sigma-Aldrich) used in this study. (b) Images of the dispersions of GN after treatment with NaClO (6 wt%) at 70°C for 0-6 h. (b) Change in the amount of GN in dispersions during treatment, estimated from the absorbance at 700 nm. GN powder consists of short stacks of graphene sheets with a few layers. To prepare dispersions, GN powder was dispersed in a bovine serum albumin (BSA) solution (CNTs = 0.5 mg/mL, BSA = 10 mg/mL) by sonication. The GN dispersion was then added to NaClO (6 wt%) to obtain a final GN concentration of 0.02 mg/mL, and incubated at 70°C for 0-6 h. The absorbance of graphene dispersions during treatment was measured at 700 nm to estimate the change

in GN concentration. The black color of the GN dispersion became colorless, and the absorbance of dispersions at 700 nm decreased to almost zero, indicating the complete degradation of GNs by NaClO solution.

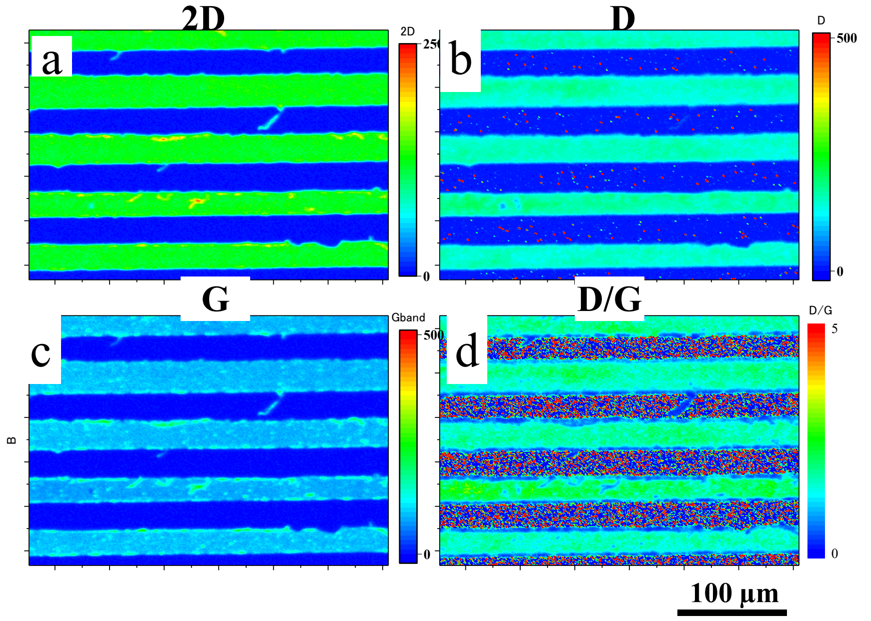

Figure SI-2. Raman mapping of large areas of patterned graphene arrays on SiO_2/Si substrate. (a) 2D band at 2690 cm^{-1}. (b) D-band at 1350 cm^{-1}; (c) G-band at 1590 cm^{-1}. (d) Ratio of D-band and G-band intensities (I_D/I_G).

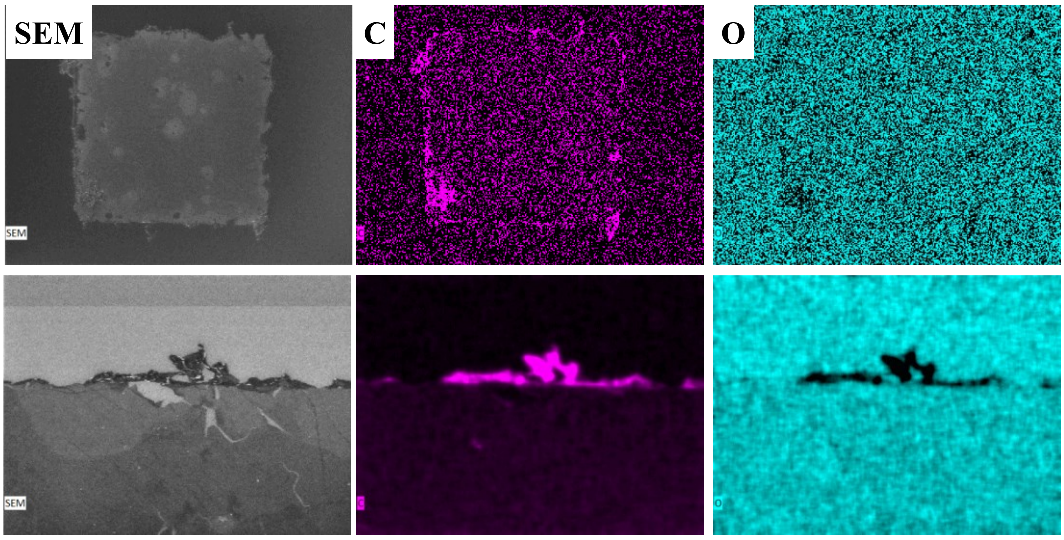

Figure SI-3. Characteristics of the structure of patterned graphene. SEM images and elementary mapping of carbon (C) and oxygen (O) are shown for a patterned graphene square on SiO_2/Si substrate.

(a)

1) $HOCl + [h\nu] \rightarrow HO\cdot + Cl^{\cdot}$

2) $OCl^{-} + [h\nu] \rightarrow O^{\cdot -} + Cl^{\cdot}$

3) $OCl^{-} + [h\nu] \rightarrow O^{3} + Cl^{-}$

(b)

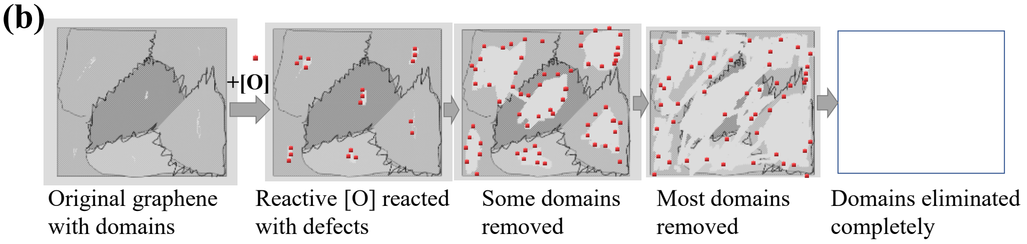

Figure SI-4. Schematic illustration of wet etching of graphene by hypochlorite and UV light. (a) Possible reaction. (b) Possible process.

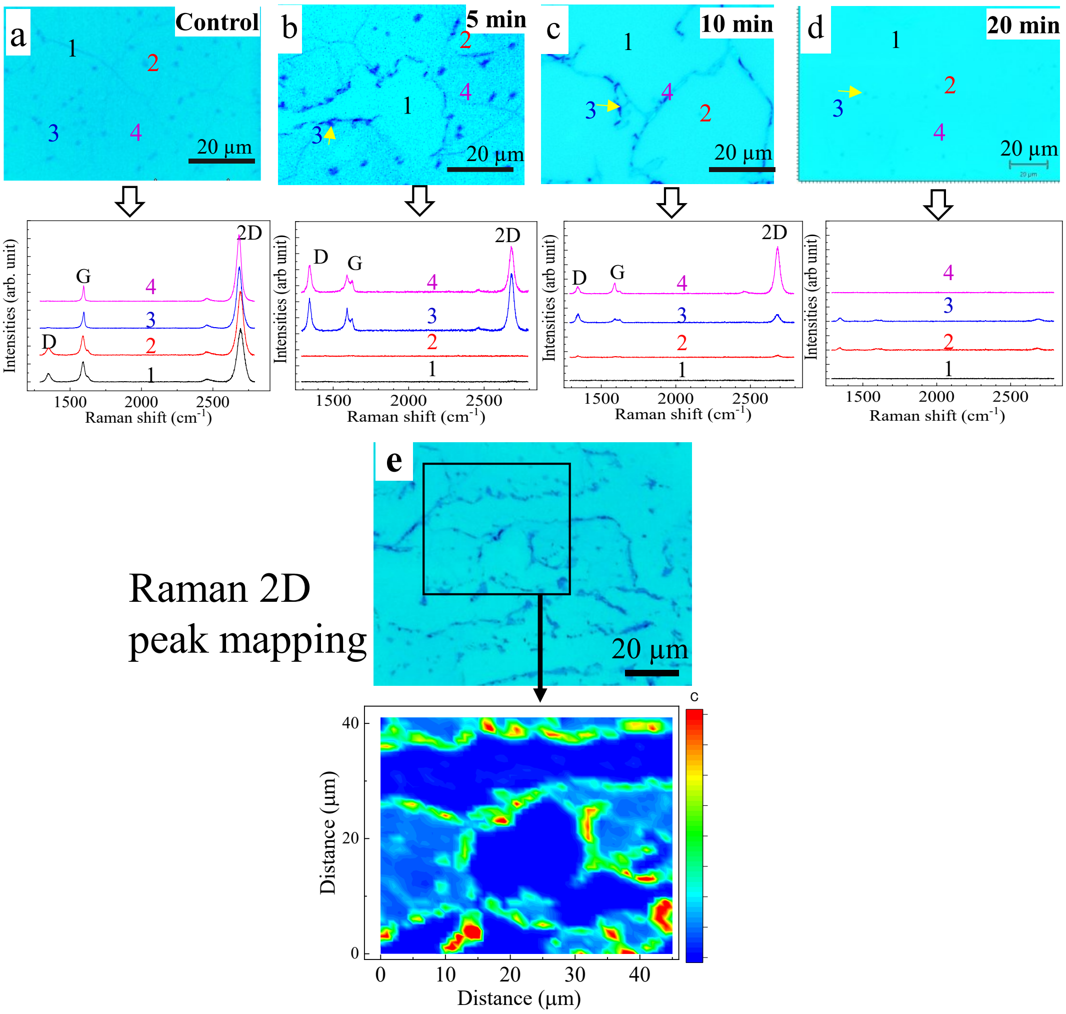

Figure SI-5. Optical microscopy images of graphene film on SiO_2/Si substrate. Images are shown before treatment (a) and after treatment with sodium hypochlorite solution (2.26 wt%) with UV light irradiation for 5 min (b), 10 min (c), and 20 min (d). Raman spectra are shown for spots (1, 2, 3, and 4) in images (a, b, c, and d), respectively. (e) Raman mapping of 2D peaks of graphene after etching for 5 min.

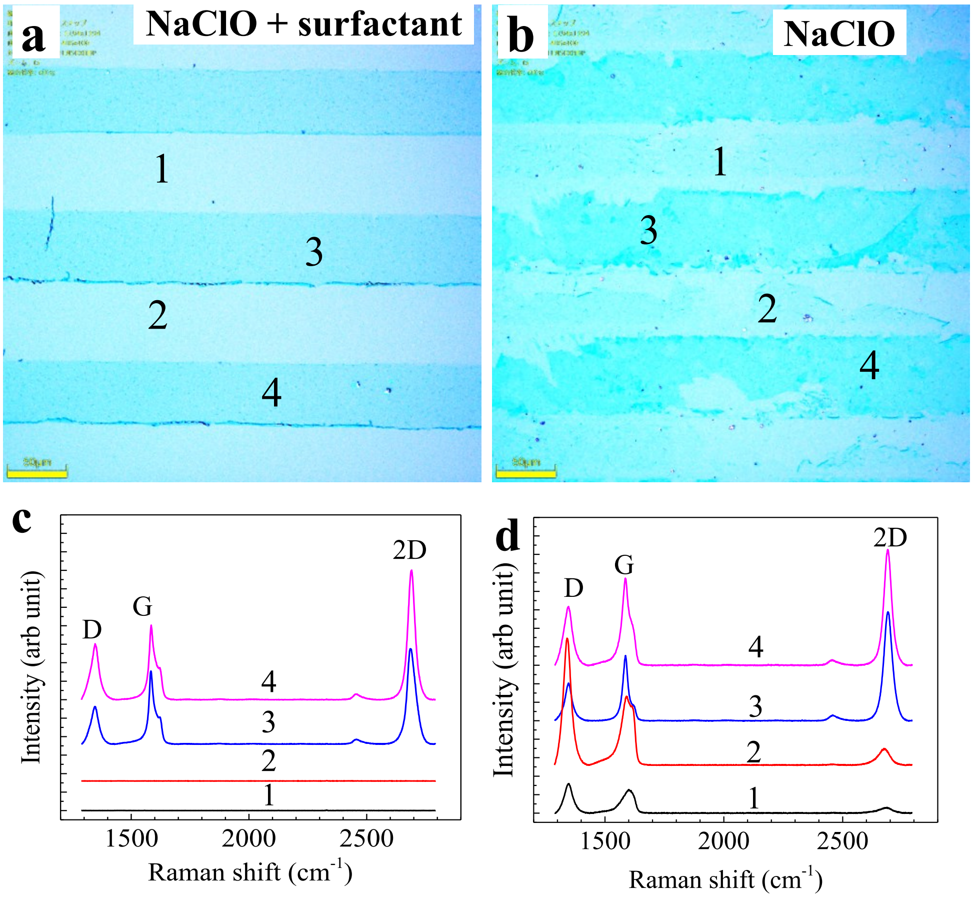

Figure SI-6. Effects of surfactant on the NaClO etching of graphene. Instead of bleach solution, sodium hypochlorite solution (6 wt%) and UV light irradiation for 5 min were applied with or without addition of a surfactant (1% N,N-dimithyldodecyamine-N-oxide). The patterning process was the same as described in the methods section. Optical microscopy images show the patterned graphene arrays obtained with (a) or without (b) surfactant. Raman spectra for spots corresponding to the patterned areas (1 and 2) or blank areas (3 and 4) in (a) and (b) are shown in (c) and (d), respectively. The results indicate that graphene was completely removed from etched areas after treatment with addition of surfactant, while some graphene patches remained after treatment without addition of surfactant.

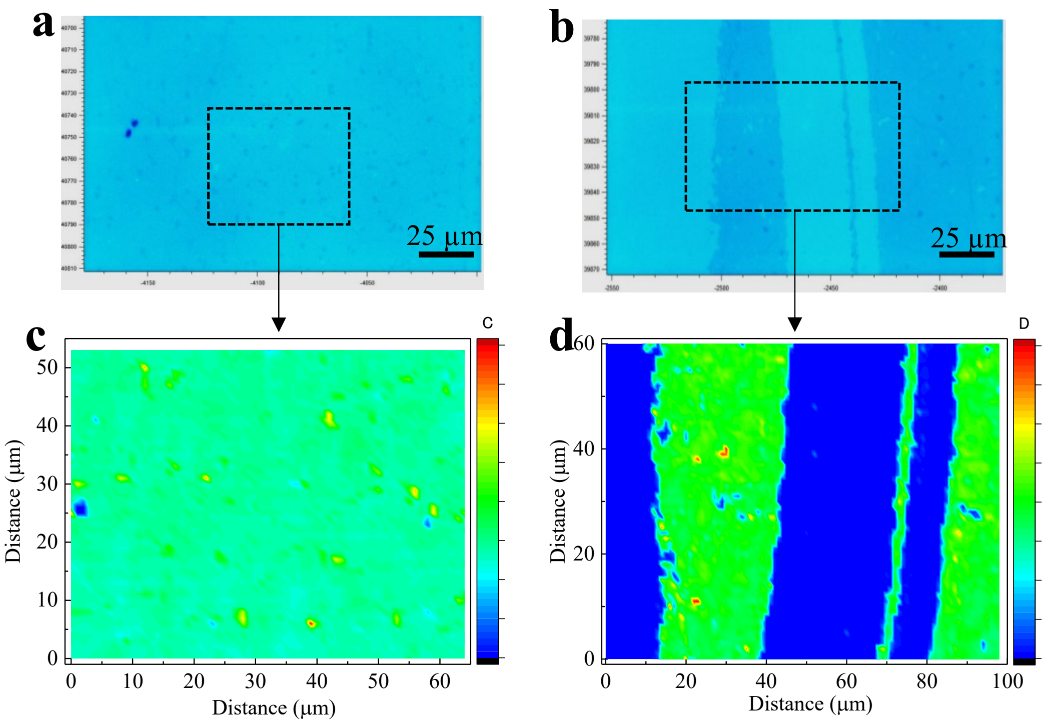

Figure SI-7. Typical patterned graphene arrays prepared using a negative photoresistor. Optical microscopy images for original graphene (a), and for graphene patterned on an Si-wafer by bleach etching (b). Raman mapping of 2D peak intensities at ~2683 cm^{-1} (c) and (d) corresponded to areas marked by black squares in (a) and (b).

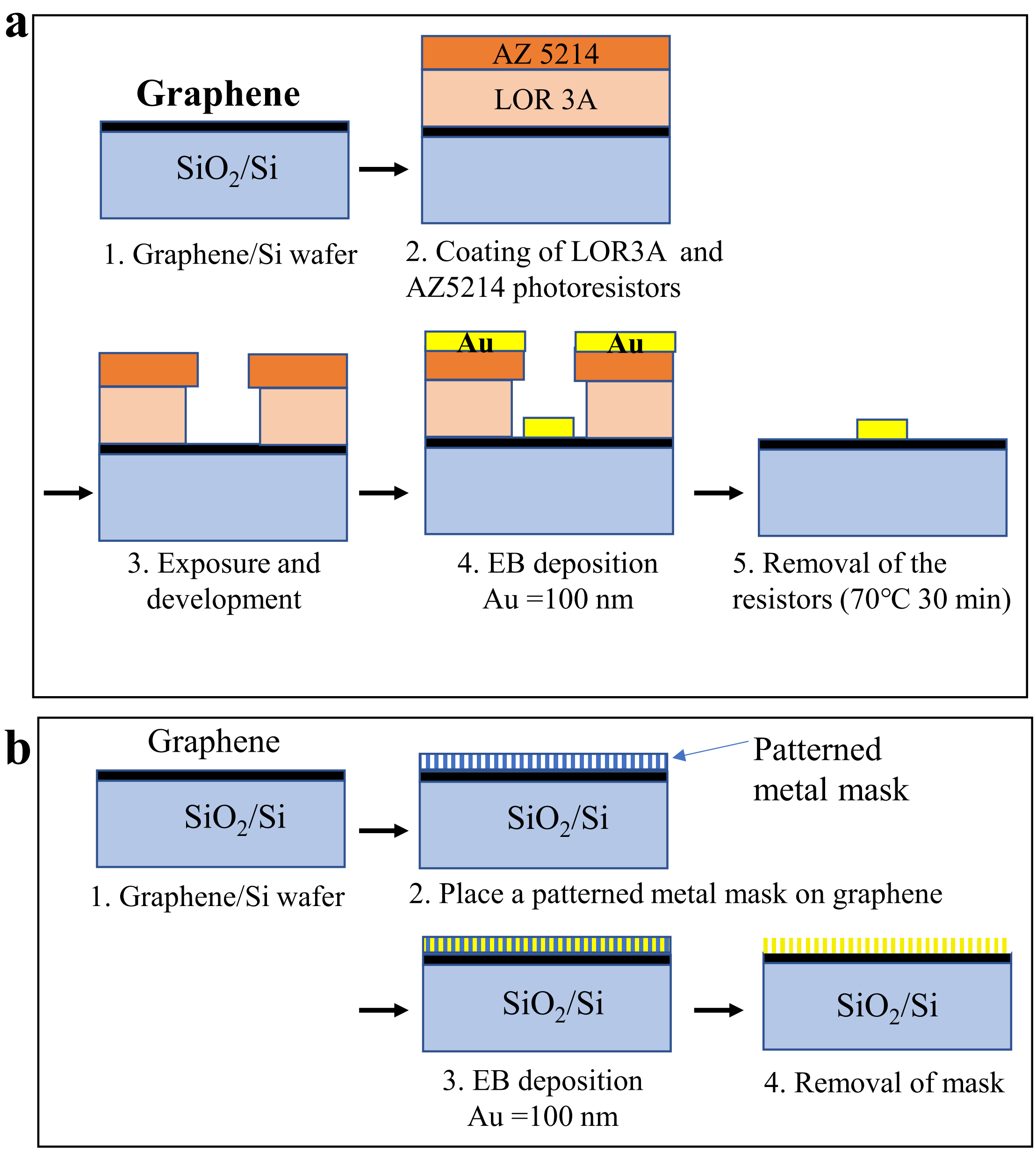

Figure SI-8. Schematic illustration of the process for preparing the patterned gold protective covering. (a) The process using photoresistor lithography. (b) The process using a metal mask.
